# Bridge Work

**Published:** 1886-10

**Authors:** L. P. Haskell


ARTICLE VI.

### BRIDGE-WORK.

### DR. L. P. HASKELL'S OPINION OF IT.

**(Written for the South Carolina Dental Society.)**

While there are occasional cases where this method is advisable, they are the exceptions. I would instance the case of the loss of a *single* tooth. It would be unfortunate for the patient, as is usually the case, to be compelled to wear a plate just to sustain that tooth; so that a tooth soldered to a bar, the ends of which could be inserted in the adjoining teeth, in gold or even cement fillings, would be perhaps the lesser evil.

But take the case of several teeth attached to two sound teeth, enclosed in gold bands—what results? The cement with which they are finally fastened in place, by constant use of the denture, is loosened and disintegrated, and works out; the secretions flow in, and the tooth is girdled with decay. Next follows the loosening of these teeth by the use of the teeth attached in mastication, so that they are found, in a few years, dangling, ready to fall out. Then there is a certain amount of uncleanliness, even in the best adjusted cases; parts that *cannot* be reached to cleanse—and finally, difficulty of repair.

The least objectionable are those cases where they are attached to roots; but here results, in a few years, at most, the loosening of the roots, in consequence of the strain upon them of an entire denture, in the act of mastication, and the patient has been to a heavy extra expense to secure the work (I have known of $500 being paid,) and now it is worthless, and must resort to the inevitable suction plate, which had better have been made at first, certainly as a matter of economy.

In a vast majority of cases where bridge-work is used, a narrow, nicely-fitting gold plate, secured by *properly-adjusted* clasps, upon the same teeth which had been permanently enclosed for bridge-work, would answer the same purpose, and could be readily removed for cleansing, and no harm done to the natural teeth. I have been in the habit of making such plates for forty years, and can testify from this long experience.

When I speak of *properly* adjusted clasps, I mean a narrow (platinum alloyed gold) clasp, nicely adjusted to the tooth, and arranged with wax upon the plate, *in the*

*mouth* (never by a plaster cast.) Then invest in the plaster and sand, and solder, attaching *only at one point*, one-eighth of an inch, or but little more, so that the clasp will be springy, and have free play. Then if this is kept clean, it will do no harm to the tooth.

The truth of the matter is, that bridge-work enables the dentist to secure large fees, regardless of the interests of patients—often twice or three times what would be charged for gold plates by the usual process.

The patient, of course, is pleased with the work, never suspecting what is in store in the near future—the loss of valuable teeth, and the final resort to a suction plate.—*Southern Dental Journal.*

